# Antifungal Activity of Crude Extract from the Rhizome and Root of* Smilacina japonica* A. Gray

**DOI:** 10.1155/2019/5320203

**Published:** 2019-07-15

**Authors:** Wei Liu, Baozheng Sun, Manman Yang, Ziyue Zhang, Xiuxiu Zhang, Tingsong Pang, Shengzheng Wang

**Affiliations:** ^1^Faculty of Pharmacy, Shaanxi University of Science and Technology, Xi'an 710021, China; ^2^Department of Medicinal Chemistry, School of Pharmacy, Fourth Military Medical University, Xi'an 710032, China

## Abstract

This study aimed to investigate the antifungal activity of hydroalcoholic extract from* Smilacina japonica ***A. Gray** (SJA) against different fungi. The minimum inhibitory concentration (MIC) for SJA was determined by the broth microdilution method. The antifungal effects of SJA against* Candida albicans* were further confirmed by cell growth test and time-kill curve test. The effects of SJA on the fungal morphology and ultrastructure were also evaluated. SJA has a broad-spectrum antifungal activity. The MICs of SJA against different fungi, including fluconazole-sensitive and -resistant* Candida albicans*, other* Candida* species, and* Cryptococcus neoformans*, ranged from 208 *μ*g/ml to 1665 *μ*g/ml. Furthermore, SJA displayed fungicidal activity against varied fungi and obviously inhibited the hyphal growth of fungi. The mechanism study revealed that the antifungal activity of SJA might be associated with its effect on the cell morphology and ultrastructure.

## 1. Introduction

With increased patients with HIV infection, receiving different immunosuppressant treatments, antineoplastic chemotherapy, transplant recipients, or using catheters or other intravenous devices, invasive fungal infections are severe infections and constantly rising in the world [1–3]. Invasive candidiasis remains the most frequent mycosis and is often cited as the fourth most common cause of bloodstream infections with significant morbidity and mortality [4]. To date, common agents used in clinic to treat invasive fungal infections are azoles (*e.g.,* fluconazole, itraconazole, voriconazole), echinocandins (*e.g.,* caspofungin, anidulafungin, micafungin), and amphotericin B. However, progressive increase in exposing to azoles and echinocandins among* Candida* isolates commonly leads to resistant strains [5, 6]. Some fungi are intrinsically resistant to certain antifungals, such as* Candida krusei* (to fluconazole) and* Cryptococcus *spp (to the echinocandins). Drug resistance in fungi worsened the already significant mortality associated with the invasive fungal infections [7]. To explore novel antifungal agents with new molecular mechanism is a valuable solution to this problem.

Traditional medicinal plants have been an important source to discover new agents or lead compounds in the history of drug research and development. A variety of herbal medicines, as* Rosa chinensis ***Jacq.***, Indigofera suffruticosa ***Mill.***, Wrightia tinctoria ***R. Br.***, Mahonia aquifolium *** Nutt.***, Glycyrrhiza glabra ***L.** and so on, were reported to exert antifungal activity [8–13]. In our screening study, we found that* Maianthemum japonicum* (**A. Gray**) LaFrankie, also called* Smilacina japonica *** A. Gray,** displayed potent antifungal and even fungicidal effect.* Smilacina japonica *** A. Gray** (SJA), locally called “PianTouQi”, is a perennial herb which distributes widely in China. The tender aerial part is edible as a wild vegetable by local people. The rhizome and root have the ability of promoting blood circulation and alleviating pain. It has long been used as folk herbs for the treatment of internal lesion caused by overexertion, asynodia, headache, rheumatism, menstrual disturbance, mastitis, bruises, carbuncle, and furuncle [14].

Previous studies have reported that SJA had several pharmaceutical effects including antibacterial, antitumor, and antioxidant activities [14–16]. However, there are few studies on its mode of antifungal action. In this context, the antifungal activity of SJA and its possible mechanisms of action were reported.

## 2. Materials and Methods

### 2.1. Strains and Media

One clinical isolate of fluconazole (FCZ) resistant* C. albicans* (103), one international calibration strain (SC5314), one American type culture collection (ATCC)-typed* Candida *strain (*C. parapsilosis *ATCC22019),* Cryptococcus neoformans* 32609,* C. krusei *ATCC2340,* C. glabrata* ATCC1182, and* C. tropicalis *2718 were utilized. SC5314 was kindly provided by William A. Fonzi (Department of Microbiology and Immunology, Georgetown University, Washington, DC).* C. krusei *ATCC2340 and* C. glabrata* ATCC1182 were kindly provided by professor Changzhong Wang (School of integrated traditional and western medicine, Anhui university of traditional Chinese medicine, Hefei, China). The other fungi strains were provided by the Changhai Hospital (Shanghai, China). Strains were cultured at 35°C under constant shaking (200 rpm) in complete liquid medium (yeast extract peptone dextrose-YPD) consisting of 1% (w/v) yeast extract, 2% (w/v) peptone, and 2% (w/v) dextrose.

### 2.2. Medicinal Material Extraction

The dried rhizome and root of SJA were bought from the fourth chain of Huaiya drug store belong to Meixian De-er-kang herbal medicine corporation in Shaanxi province with supply certificate. Approximate 5 g of dried and smashed SJA was extracted with an excess 100 ml of 75% ethanol, then the extract was filtered. After removing the solvent by rotary evaporation under reduced pressure at 50°C, the residue was dissolved with DMSO to approximately 0.333 g/ml SJA. The final extract was dark brown liquid and stored at -20°C for further use.

### 2.3. Antifungal Susceptibility Test

The antifungal susceptibility test was performed on all strains according to broth microdilution method as described previously [17].* C. parapsilosis* ATCC22019 was used as a quality control strain and tested in each assay. FCZ was used as a positive drug control. The initial concentration of fungi suspended in RPMI 1640 medium was about 10^3^ cells/ml, and the initial concentration ranged from 0.125 to 64 *μ*g /ml for FCZ and 6.5 to 3330 *μ*g /ml for SJA. The 96-well plates were incubated at 35°C for 24 h, 48 h or 72 h. The minimum inhibitory concentrations (MICs) were determined by optical density. Each strain was tested in triplicate.

### 2.4. Checkerboard Microdilution Assay

Assays were performed according to the above test 2.3. The combination concentrations ranged from 0.125 to 64 *μ*g/ml for FCZ and 26 to 208 *μ*g/ml for SJA. The fractional inhibitory concentration (FIC) index (FICI) was calculated by the following equation: FICI = FIC A + FIC B, where FIC A is the MIC of the combination/the MIC of drug A alone, and FIC B is the MIC of the combination/the MIC of drug B alone. Synergy was defined as an FICI value of ≤0.5, while antagonism was defined as an FICI value of >4, and the addition was defined as an FICI value of 0.5 < FICI≤1. An FICI result between 1 and 4 was considered indifferent.

### 2.5. Cell Growth Test

Each fungus was prepared at the starting inoculum of 10^6^ cells/ml in glass tubes. Different concentrations of SJA (208 *μ*g/ml, 416 *μ*g/ml, 832 *μ*g/ml and 3330 *μ*g/ml) and FCZ were added into tubes. After incubation with agitation at 35°C for 24 h or 48 h, pictures were captured [18].

### 2.6. Time-Kill Curve Studies

Different fungi strains were prepared at the starting inoculum of 10^5^ cells/ml. The concentrations were 104 *μ*g/ml, 208 *μ*g/ml, 416 *μ*g/ml or 832 *μ*g/ml for SJA, and DMSO comprised < 1% of the total test volume. At predetermined time points (0 h, 4 h, 8 h, 12 h,16 h, 24 h and 48 h) after incubation with agitation at 35°C, a 100 *μ*l aliquot was removed from every solution and serially diluted 10-fold in sterile water. A 100 *μ*l aliquot from each dilution was spread on the sabouraud dextrose agar plate. Colony counts were determined after incubation at 35°C for 48 h. Fungicidal activity was defined as a ≥3 log_10_ reduction from the starting inoculums [19].

### 2.7. Hyphal Growth Test

*C. albicans *SC5314 cells were incubated at 37°C in RPMI 1640 for 3 h in the absence or in the presence of different concentrations of SJA (416 *μ*g/ml, 832 *μ*g/ml) or FCZ (0.5 *μ*g/ml). All cells were viewed by light microscopy to assess hyphal formation [20].

### 2.8. Cell Membrane Permeability

Membrane permeabilization of* C. albicans* was detected according to the study [21]. Briefly,* C. albicans *SC5314 (10^7^ cells/ml) was incubated with 5 mM calcein acetoxy-methyl ester (Fanbo biochemicals, China) for 2 h. Then the cells were washed three times and* C. albicans *(10^6^ cells/ml) was transferred into tubes. After treating with or without SJA (416 *μ*g/ml, 832 *μ*g/ml) for 3 h, the cells were washed three times and about 20,000 cells were acquired for flow cytometry analysis (Maflo Astrios flow cytometer).

### 2.9. Transmission Electron Microscopy (TEM)

After treating with or without SJA (416 *μ*g/ml or 832 *μ*g/ml),* C. albicans* SC5314 was washed in PBS and fixed in 3 ml fixative solution (sodium cacodylate buffer, pH 7.2, containing 4% polyoxymethylene) for 24 h at 4°C. Then, the samples were washed with saline and postfixed with 1% phosphotungstic acid for 90 minutes. The fixed cells were dehydrated through a graded series of ethanol and embedded with EPON-812. Ultrathin sections were prepared and observed after double staining with uranium and lead under a transmission electron microscope (HITACHI H-800, Japan) [22].

### 2.10. Statistical Analysis

The two-tailed Student's t-test was used for analysis of two groups. Statistical significance was set at a p-value in the figures as: *∗*, P < 0.05.

## 3. Results

### 3.1. Fungi Growth Was Inhibited by SJA

**Figure 1:**
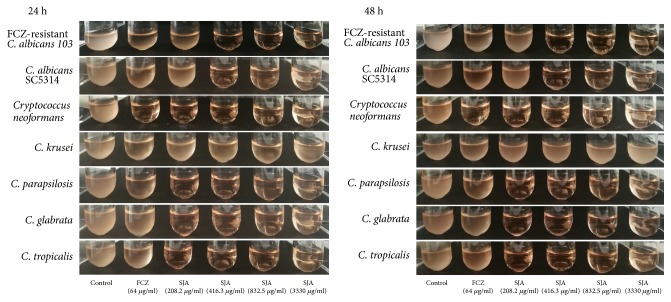
SJA inhibits the growth of part fungi. Different fungi strains with an initial inoculum of about 10^6^ cells/ml were exposed to 64 *μ*g/ml FCZ or different concentrations of SJA (208 *μ*g/ml, 416 *μ*g/ml, 832 *μ*g/ml and 3330 *μ*g/ml). Pictures were captured after 24 h and 48 h of incubation.

The results of the antifungal susceptibility test were summarized in Table 1. SJA displayed a broad-spectrum antifungal activity. The MICs of SJA against different fungi, including FCZ-sensitive and -resistant* C. albicans*, other* Candida species* and* Cryptococcus neoformans*, ranged from 208 *μ*g/ml to 1665 *μ*g/ml at 24 h or 48 h. To further visualize the activity of SJA against these fungi, we evaluated the growth of fungi after incubation with different concentrations of SJA for 24 h and 48 h. SJA at 416 *μ*g/ml, 832 *μ*g/ml and 3330 *μ*g/ml displayed potent growth inhibition activity against all the tested fungi except* C. krusei* (Figure 1). In addition, SJA at 208 *μ*g/ml also exhibited significant growth inhibition against part fungi (*Cryptococcus neoformans, C. glabrata, C. parapsilosis *and* C. tropicalis*).

To evaluate the interaction of SJA with antifungal drug FCZ, checkerboard microdilution assays were tested. As shown in Table 2, synergisms were observed for* Cryptococcus neoformans* and* C. krusei*, while additions were found for FCZ-resistant* C. albicans *103,* C. parapsilosis* and* C. tropicalis. *Indifferences were observed for* C. albicans* SC5314 and* C. glabrata.*

**Figure 2:**
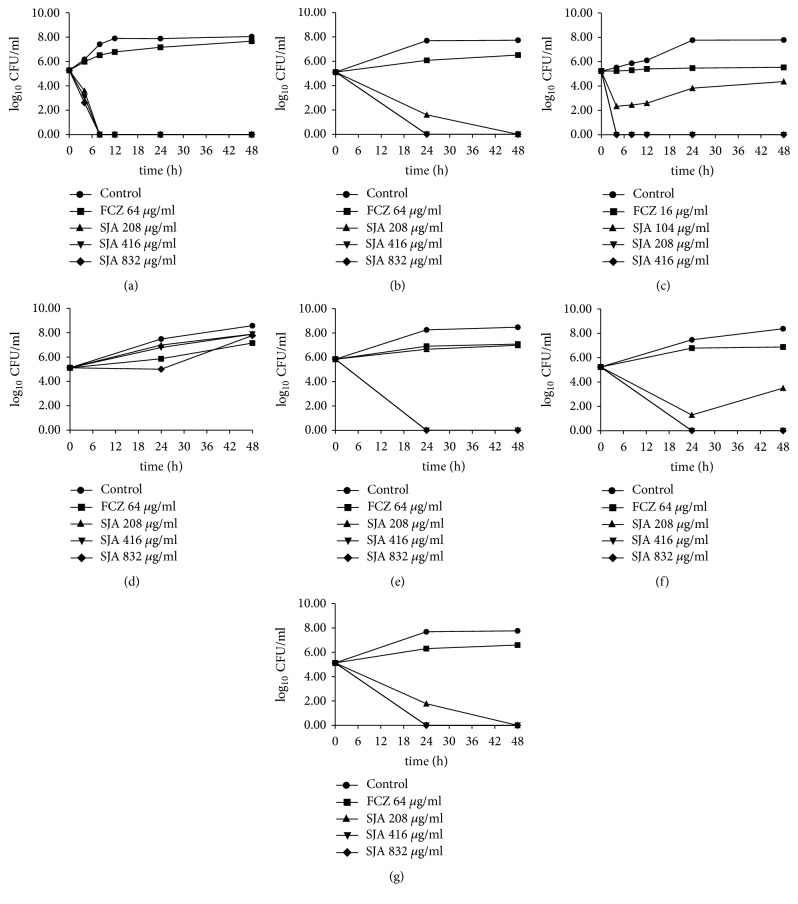
Time kill curves of various fungi treated with different concentrations of SJA and FCZ. FCZ-resistant* C. albicans* 103 (a),* C. albicans* SC5314 (b),* Cryptococcus neoformans* (c),* C. krusei *(d),* C. parapsilosis* (e),* C. glabrata* (f) and* C. tropicalis* (g) were treated with different concentrations of SJA (104 *μ*g/ml, 208 *μ*g/ml, 416 *μ*g/ml and 832 *μ*g/ml) for 48 h. Aliquots were obtained at the indicated time points and serially dilutions were spread on agar plates. Colony counts were determined after 48 h incubation.

To evaluate whether SJA had fungicidal activity against fungi, time-kill curve studies were performed. As shown in Figure 2, SJA displayed potent fungicidal effect against different fungi except* C. krusei, *which was consistent with the results of cell growth test. SJA at 208 *μ*g/ml displayed fungicidal effect against FCZ-resistant* C. albicans* 103 and led to a decrease of 1.68-log_10_ Colony-forming units (CFU) /ml at 4 h compared with the initial inoculum concentration (Figure 2(a)). Moreover, SJA at 208 *μ*g/ml, 416 *μ*g/ml and 832 *μ*g/ml resulted in a complete cell-killing at 8 h. For* C. albicans *SC5314 (Figure 2(b)). SJA also displayed fungicidal effect. SJA at 208 *μ*g/ml yielded a 6.1-log_10_ CFU/ml reduction compared with the control at 24 h. More specifically, SJA at 208 *μ*g/ml, 416 *μ*g/ml and 832 *μ*g/ml exhibited a complete cell-killing at 48 h. For other fungi (*Cryptococcus neoformans, C. glabrata, C. parapsilosis *and* C. tropicalis*), both 416 *μ*g/ml and 832 *μ*g/ml SJA resulted in a complete cell-killing at 24 h, and the densities of fungi cells were all reduced to zero. These results confirmed that SJA had excellent fungicidal activity against varied fungi.

### 3.2. Hyphal Growth of Fungi Was Inhibited by SJA

**Figure 3:**
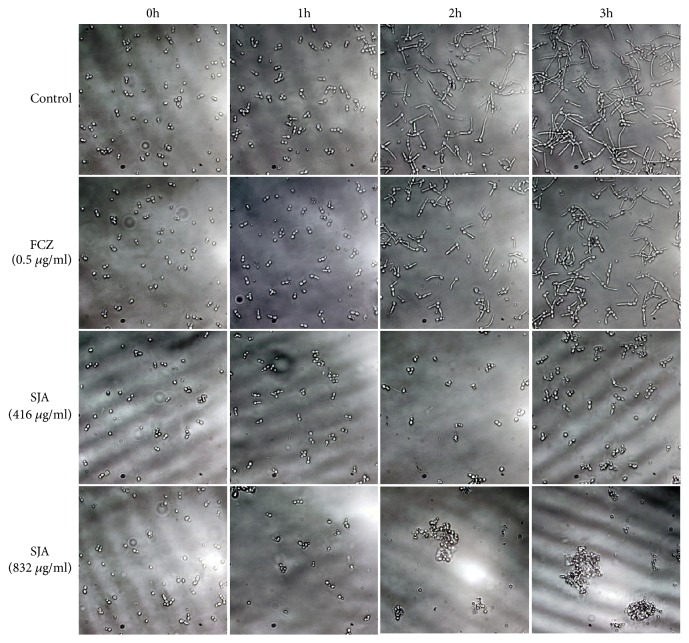
SJA inhibits the hyphal growth of fungi. The* C. albicans* SC5314 cells were incubated in RPMI 1640 liquid medium at 37°C for 3 h in the absence or presence of different concentrations of SJA (416 *μ*g/ml or 832 *μ*g/ml) or FCZ (0.5 *μ*g/ml). The hyphal development was viewed by light microscopy and photographed after 0 h, 1 h, 2 h and 3 h of incubation.

Fungi like* C. albicans* can grow in three types of morphological forms including yeast, pseudohyphae, and hyphae. During the early stage of infection, yeast fungi become hyphae form to penetrate human epithelial and endothelial cells and cause the damage to them [23, 24]. In addition, to resist standard antifungal treatments, fungi can form biofilms which contain hyphae [25]. Therefore, the activity of SJA on the growth of fungal hyphae was determined. In the blank control group (Figure 3),* C. albicans* showed elongated hyphae forms after 3 h incubation. After treating with SJA at 416 *μ*g/ml or 832 *μ*g/ml, most of the* C. albicans* did not germinate and appeared as yeast form. More interestingly,* C. albicans* progressively aggregated and became clumps after treating with 832 *μ*g/ml of SJA for 2 h and 3 h.

### 3.3. The Cell Size and the Membrane Permeability of Fungi Were Increased by SJA

**Figure 4:**
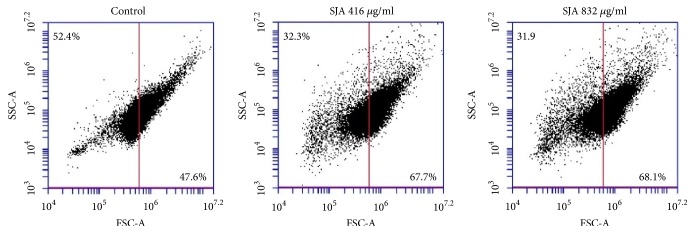
Change of cell size (forward scatter-side scatter) in the presence of SJA.* C. albicans* SC5314 were treated with or without SJA (416 *μ*g/ml to 832 *μ*g/ml) for 3 h. Then the cells were analyzed by flow cytometry.

Flow cytometry analysis (side scatter [SSC]-forward light scatter [FSC]) revealed that* C. albicans* SC5314 treated with SJA (416 *μ*g/ml or 832 *μ*g/ml) exhibited a significant increase in cell size, as evidenced by the increase in forward light scattering (Figure 4).

**Figure 5:**
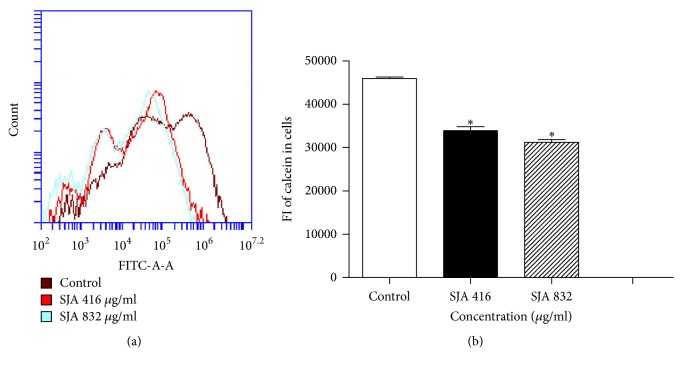
The effect of SJA on cell membrane permeability. Candida cells (10^7^ cells/ml) were cultured in the presence of 5 *μ*M calcein acetoxymethylester for 2 h. After washing three times, the cells were cultured in the presence or absence of SJA (416 *μ*g/ml to 832 *μ*g/ml) for 3 h. Cellular fluorescence intensities of calcein were analyzed by flow Cytometry. Data are means ± SD of triplicates of one representative experiment of three. *∗*, P < 0.05, Student's t-test.

Calcein AM is a non-fluorescent, hydrophobic compound that easily permeates intact, live cells. Once into the cytoplasm of cells, calcein AM is hydrolyzed by cytoplasmic esterases, producing membrane-impermeable, hydrophilic and strongly fluorescent calcein which can be well-retained in intact cell cytoplasm. After incubation with calcein AM, the cellular fluorescence of calcein was detected by flow cytometry to evaluate the effect of SJA on the cell membrane permeabilization. Result displayed that cellular fluorescence of calcein was markedly decreased by treatment of* Candida* cells with different concentrations of SJA (416 *μ*g/ml or 832 *μ*g/ml) compared with the blank control cells (Figure 5). The results indicated that SJA interrupted the* Candida* cell membrane and increased the cell membrane permeability.

### 3.4. The Ultrastructure of Fungi Was Damaged by SJA

**Figure 6:**
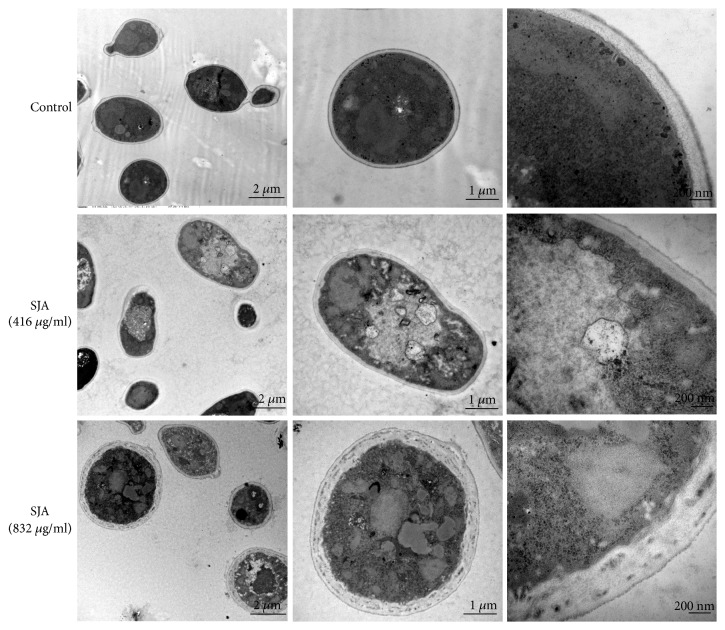
TEM results of* Candida *cells treated with or without SJA (416 *μ*g/ml or 832 *μ*g/ml).

In order to explore the antifungal mechanism of SJA, the ultrastructure of* C. albicans* was monitored by TEM. As shown in Figure 6, the control* Candida* cells have a uniform central density and an intact cell wall. After treatment of SJA at 416 *μ*g/ml or 832 *μ*g/ml, the outer layer of the cell wall became thinner, and the middle layer became thicker. Some abnormal shapes of the vacuoles were also observed. In addition, reduced cytoplasmic density and damaged membranes could be observed. The TEM results indicated that SJA caused obvious damage on the ultrastructure of* C. albicans* including cell wall and membrane structures.

## 4. Discussion

Fungal diseases cause considerable morbidity and mortality globally and elevate health care costs [26]. The paucity of effective antifungal agents and emergency of drug resistance prompted researchers to develop novel antifungal agents [7]. Natural products are a valuable source for drug discovery, whether in their nascent form as original templates for structure-optimizing for more effective and safe derivatives. About 80% of marketed antibiotics used clinically are derived from natural products [27, 28]. Current antifungal drugs like echinocandins and amphotericin B are also developed from natural resources. A variety of natural products, as peptides, saponins, flavonoids, alkaloids, essential oils, and so on, have been found to possess antifungal activity [29–33]. As a traditional Chinese medicine, SJA has various therapeutic effects. However, to the best of our knowledge, there was no investigation on the action of SJA against fungi.

In this study, we demonstrated that SJA had antifungal activity against different fungi including* Candida species* and* Cryptococcus neoformans *(with MICs ranging from 208 *μ*g/ml to 1665 *μ*g/ml). In light of the definitions of fungicidal effect through time-kill curve tests, a fungicidal effect was defined as a reduction in ≧3log_10_CFU /ml compared to initial inoculum [34]. Therefore, SJA was considered fungicidal. Previously, the antifungal effect of steroidal saponins extracted from the genus* Smilacina* called* Smilacina atropurpurea *(Franch.)** Wang et Tang** (Convallariaceae) was studied by Ying Zhang et al. [35]. Among eight saponins, they found that astropuroside B and F exhibited fungicidal activity against* C. albicans, C. glabrata, Cryptococcus neoformans *and* Aspergillus fumigates *with MFCs≤20 *μ*g/ml except* C. krusei*, while dioscin was selectively active against* C. albicans* and* C. glabrata* (MFC≤5.0 *μ*g/ml) [35]. Compared to* Smilacina atropurpurea*, the extract of SJA exhibited similar fungicidal effect, but it had a broader antifungal spectrum. According to previous reports on the constituents of the two plants, there are differences in the chemical structures of saponins isolated from* Smilacina atropurpurea *and SJA [14, 36–38]. In addition, the report by Ying Zhang et al. also suggested that not all saponins had antifungal activity [35]. Therefore, our group will perform more studies to explore the exact antifungal ingredients of SJA in collaboration with the other laboratory.

Hyphae play an important role in the fungal infection and biofilm formation. SJA was able to suppress the hyphal growth of* C. albicans.* Besides, through the analysis of cellular fluorescent calcein by flow cytometry, we demonstrated that SJA could increase the membrane permeability. The yeast-to-hypha inhibition and the membrane-perturbation effect of SJA are akin to the other saponins like saponin extract from rhizomes of* Dioscorea panthaica ***Prain et Burk **and tea saponin [30, 39].

TEM results showed that SJA obviously damaged the ultrastructure of fungi including the membrane structures and cell wall. Lesions were observed on membrane structures, including the cytoplasmic membrane and organelle membranes such as nucleus and mitochondria. The membrane damage of SJA may contribute to the increased membrane permeability and cell size of fungi. SJA also exhibited a harmful effect on the cell wall, such as the outer layer of the cell wall became thinner, and the middle layer became thicker. Cell wall plays a crucial role in the fungal commensalism and infection [40]. It offers mechanical strength and acts as a barrier, thus protecting the fungus from the hostile environment. Besides, the cell wall glucan plays an important role in the yeast-to-hypha transition [41]. This suggests the hyphal growth inhibition of SJA may be related to its damage on the ultrastructure like cell wall. The harmful effects on the membrane and cell wall of SJA are similar to the* Solanum chrysotrichum* saponin SC-2 and saponin extract from rhizomes of* Dioscorea panthaica ***Prain et Burk **[30, 42].

## 5. Conclusion

The present study connoted that SJA has strong fungicidal activity against different fungi such as* Candida *species and* Cryptococcus neoformans*. SJA could also inhibit the yeast-hypha transition and increase membrane permeability and cell size of* C. albicans*. The antifungal effects might be through membrane and cell wall damage. As a traditional Chinese medicine, SJA represents a promising herbal medicine for the discovery of novel antifungal compounds. The exact antifungal ingredients and action mechanisms are under investigation in our laboratories.

## Figures and Tables

**Table 1:** Antifungal activity of SJA against different fungi (*μ*g/ml) ^*a*^.

fungi	MIC (*μ*g/ml)			
	FCZ^*b*^		SJA	
	24 h	48h	24 h	48h
FCZ-resistant *C. albicans *103	>64	>64	416-832	416-832
*C. albicans* SC5314	0.5-1	1	416-832	416-832
*Cryptococcus neoformans*	1-2	2	208	208-416
*C. krusei *	>64	>64	1665	1665-3330
*C. parapsilosis*	2	2	416	416-832
*C. glabrata*	>64	>64	416	208-416
*C. tropicalis *	>64	>64	208-416	416-832

^*a*^ SJA, *Smilacina japonica ***A. Gray**; ^*b*^ FCZ, fluconazole.

**Table 2:** Activity of SJA alone and in combination with FCZ against different fungi (*μ*g/ml) ^*a*^.

fungi	Alone		Combination		FIC index for combination	Mode of interaction
	FCZ^*b*^	SJA	FCZ^*b*^	SJA		
FCZ-resistant *C. albicans *103	>64	416	64	208	1.000	addition
*C. albicans* SC5314	1	416	1	26	1.125	indifference
*Cryptococcus neoformans*	2	208	0.5	26	0.375	synergism
*C. krusei*	>64	1665	8	208	0.188	synergism
*C. parapsilosis*	2	416	1	26	0.563	addition
*C. glabrata*	>64	416	>64	208	1.500	indifference
*C. tropicalis*	>64	416	64	208	1.000	addition

^*a*^ SJA, *Smilacina japonica ***A. Gray**; ^*b*^ FCZ, fluconazole.

## Data Availability

The data used to support the findings of this study are available from the corresponding author upon request.
